# Benefit of a topical slimming cream in conjunction with dietary advice

**DOI:** 10.1111/j.1468-2494.2010.00630.x

**Published:** 2011-08

**Authors:** B Escudier, C Fanchon, E Labrousse, M Pellae

**Affiliations:** * L'oréal Recherche188 rue Paul Hochart, 94550 Chevilly Larue; † Médecin nutritionniste, Hopital BichatParis, France

**Keywords:** cellulite, cosmetics, evaluation, nutritional balance, slimming cream

## Abstract

**Synopsis:**

The aim of this study was to determine how worthwhile it would be to combine a newly developed topical slimming product with customized dietary habits not based on calorie restriction, so as to improve the cellulite appearance of the skin. At the beginning of the study, a nutritionist recorded the dietary habits of each participant and gave recommendations to each of them according to their food consumption. The chosen methodology was a right/left comparison, one thigh and hip being treated with the new topical slimming product and the other one left untreated to serve as a random control. Objective evaluations were performed by blind assessors. Control of food intake improved the cellulite score after 4 weeks when compared with the base value, but this reduction was significantly greater and earlier on the treated side than on the untreated side, indicating an objective additional benefit derived from the new slimming cream. This result corroborated the slimming effect assessed by measurement in centimetres of the circumference of the upper thighs and the reconstructed volume of the thigh between two fixed horizontal slices. Furthermore, skin tonicity, a major component of cellulite visibility, was also significantly improved on the treated side after only 2 weeks.

This new topical cream thus enhances the benefit of a dietetic control for the treatment of the visible aspect of cellulite on the skin.

**Résumé:**

Cette étude avait pour objectif d’étudier l'intérêt éventuel d'associer une nouvelle crème amincissante à des conseils nutritionnels personnalisés, non basés sur la restriction calorique mais sur un meilleur équilibre alimentaire afin d'améliorer l'aspect cellulitique de la peau. Au début de l’étude un médecin nutritionniste a enregistré les habitudes alimentaires de chaque sujet et a donné des recommandations à chacun en fonction de leur consommation alimentaire. La méthodologie contrôlée en hémi corps, gauche/droite a été choisie, une cuisse, une fesse ayant été traités par le produit à l’étude, l'autre partie du corps étant restée sans traitement selon la randomisation. Les évaluations objectives ont été réalisées par des investigateurs en aveugle. Le contrôle des aliments a permis une réduction du score de cellulite après 4 semaines mais cette réduction est statistiquement plus importante et plus rapide du côté traité avec le produit topique que du côté non traité, ce qui correspond à un effet additionnel objectivé du produit topique. Ce résultat est conforté par l'effet amincissant évalué par la mesure centimétrique de la circonférence de la partie supérieure de la cuisse et l’évolution du volume reconstruit de la cuisse entre deux niveaux. De plus la tonicité de la peau, composante importante de la visibilité de la cellulite, est améliorée significativement dès 2 semaines du coté traité;

Cette nouvelle crème apporte donc un bénéfice complémentaire au contrôle diététique pour le traitement de la cellulite.

## Introduction

Cellulite is a very frequent and complex condition that affects 85% of post-puberty female subjects [1]. It is, irrespective of body weight, more prevalent in women than in men [2], and it is more common in Caucasian than in Asiatic women [3]. The ‘orange peel’ appearance is one of the women's main complaints, as it is clinically visible, according to severity, after or even without pinching. Even though its etiopathogenesis is not yet fully understood, many authors agree that cellulite has multifactorial – structural, genetic and endocrinological – causes. It results from a complex series of inflammatory events involving both the subcutaneous fatty tissues and the dermis [1]*.* The events appear to vary chronologically according to the main contributing factors: hydrophilic, vascular, structural or inflammatory. It may at first involve local interstitial oedema caused by increased permeability in local capillaries and veinlets, which with repetition leads to the inflammation of the reticular fibres [4]. A local increase in glycosaminoglycans in the ground substance of the dermis could explain the retention of an excess of fluid in the dermal and subcutaneous tissue [5] and could lead to the appearance of mattress skin [6]. Cellulite can affect individuals regardless of their body mass index (BMI), but unfortunately research is scant and dissention and controversy are common [7]. Good diet and physical condition do not uniformly prevent the development of cellulite [8], and in fact, the effect of caloric restriction on cellulite appearance is still a matter of debate. It has been established for a long time that weight loss is rarely followed by cellulite reduction, and one recent study [9] has provided information on the great variability of responses after weight loss programmes. Whereas it shows some positive effect for the group with a large excess of body mass and a relatively high cellulite severity score at the outset, the study indicates a worsening cellulite for the group with the smaller initial BMI. Moreover, it is well known that the skin loosens after weight loss and this can adversely affect skin dimpling [10]. Occasional dieting or continuous low-calorie dieting may certainly have different effects on femoral adipocyte size and α2 adrenergic-receptor density and sensitivity. [11].

In this study, we focused on the impact of changes in food habits rather than on calorie restriction. A leaflet containing advice by a hospital nutritionist doctor encouraged eating differently, with a better choice of food (good food habits), but without limitation on the amount of food consumed. The objective was to see whether there was an additional effect on the cellulite aspect of the skin when combining this ‘well- balanced food regimen’ with a new local topical treatment.

## Subjects and methods

### Inclusion criteria

Fifty non-obese women 18–45 years of age (mean age 32) with a cellulite severity score of at least 2 on the L’ Oréal Cellulite Chart® (scoring from 0 to 4) and a BMI between 20 and 27, who wished to slim down and improve the cellulite appearance of their skin, were enrolled in this monocentric randomized right/left comparison study. They were included after having signed informed consent. The objective was to evaluate the effectiveness of the new slimming cream in conjunction with a balanced but not low-calorie diet. To determine their nutritional profile, the recruited women had to answer 7 days before inclusion a nutritional questionnaire composed for this purpose by a hospital nutritionist doctor. After analysis of the answers on the types of foods consumed and their frequency per day or per week, and taking into account the behaviour and lifestyle of each study subject, the nutritionist doctor determined their food profile (‘sweet tooth’ or ‘salt tooth’). Recommendations were then issued to rebalance their energy supply based on their profile. It is worth noting that the balance has to be maintained over several days.

The test product containing 5% caffeine and a flavonoid-rich *Nelumbo nucifera* extract was to be applied twice daily for 4 weeks to the thigh and hip on one side (left or right) according to the randomization list. On each of the following three visits, correct application of the product and observance of the food recommendations were checked. The study was conducted between April and June.

### Efficacy evaluation/time of measurement

Efficacy was assessed through different methods by blind assessors in standardized conditions:

1. Cellulite Clinical Score according to the L'Oréal Cellulite Chart® on each thigh with a basic visual evaluation (without tissue mobilization) and after a standardized pinching to evaluate the ‘orange peel appearance’ before the start (T0) and after 1, 2 and 4 weeks of treatment.
2. Dermal Torquemeter (DTM®) to evaluate skin tonicity (Ur) on each thigh at T0, T2 and T4 weeks of treatment.
3. Circumference measurement in centimetres on the upper part of each thigh before the start (T0) and after 1, 2 and 4 weeks of treatment.
4. Reconstructed volume of the thigh, hip and buttock between two fixed horizontal slices on both sides, with 3D Fringe projection and 3D reconstruction images at T0 and T4 weeks of treatment.

## Results

All 50 subjects completed the study. Their mean BMI was 24 kg per square metre at baseline visit and 23.8 at final visit. A slight but significant reduction in the mean weight was obtained after 1, 2 and 4 weeks of treatment when compared with the baseline value (63.8 kg at baseline and 63.4 after 4 weeks).

### Cellulite clinical score without pinching

**Figure 1:**
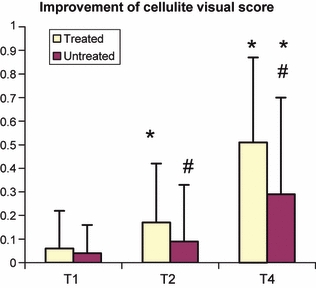
Cellulite without pinching. *Significant vs. baseline value (*P* < 0.05). ^#^Significant vs. ‘treated’ side (*P* < 0.05).

When compared with the baseline values, the clinical score showed a significant decrease over time only on the treated side at T2W, with a significantly higher reduction between T0 and T2W for the topically treated side than for the untreated side (*P* < 0.001). At T4W, there was a reduction in the clinical score on both sides vs. baseline values, but this reduction was statistically greater on the topically treated side than on the untreated side (*P* = 0.006) (Fig. 1). This means that the dietary advice contributed a slight but significant benefit to the cellulite score, whereas on the topically treated side an additional beneficial effect on the ‘orange peel’ visibility was obtained.

### The cellulite clinical score after pinching

**Figure 2:**
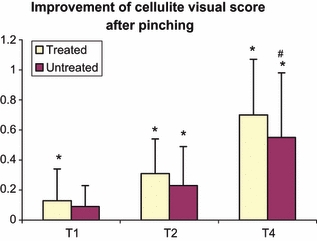
Cellulite after pinching. *Significant vs. baseline value (*P* < 0.05). ^#^Significant vs. ‘treated’ side (*P* < 0.05).

This evaluation also proved an additional beneficial effect of the topical treatment, because the difference in the evolution of the score between T0 and T4 weeks was highly significant in favour of the topically treated side (*P* = 0.006) at T4 weeks (Fig. 2).

### DTM measurements

Skin tonicity was assessed by the Ur parameter, which increased significantly on the treated side only, already after 2 weeks of topical treatment, with a significant difference vs. the untreated side (*P* = 0.006 at T2W and *P* = 0.039 at T4W, respectively).

### Circumference measurements in centimetres

Measurement of circumference in centimetres showed a significant reduction at T2W of the upper part of thighs both treated and untreated, showing no significant difference between thighs. But at T4 weeks of treatment, the evolution of the upper part of the thigh was significant only for the treated side, and the difference between the treated and untreated side was significant (*P* = 0.037) (Table I).

**Table I:** Circumference measurements

Thigh circumference in centimetres (Upper part)	Topically treated side	Topically untreated side	Δ (Ti − T0) treated side − (Ti − T0) untreated side
Δ (T1W − T0)	−0.08 ± 0.52 (NS)	−0.08 ± 0.52 (NS)	(NS)
Δ (T2W − T0)	−0.27 ± 0.58 (S)	−0.21 ± 0.58 (S)	(NS)
Δ (T4W − T0)	−0.33 ± 0.77 (S)	−0.18 ± 0.78 (NS)	*P*= 0.037 (S)

### 3D fringe projection

This measurement gives volume reconstruction of thigh, buttock and hip between two fixed horizontal slices. It showed a significant evolution at T4W for the treated thigh, with a significant difference between treated and untreated sides (*P* = 0.012). For the buttocks and hips, similar results were obtained, with a significant difference between treated and untreated sides (*P* = 0.004 and *P* = 0.0008, respectively). These results in accordance with the centimetre circumference evolution in favour of the treated side confirm the additional beneficial effect of the topical slimming cream.

Furthermore, at the end of the study, the women were asked to judge their skin on the thigh and buttock, and most of them noticed firmer, smoother, softer and suppler skin – improved skin quality – on the topically treated side.

## Discussion

We have presented a study combining ‘good dietary recommendations’ and application of a topical product to the cellulite area and have shown a beneficial effect of this combination. The nutritional advice alone was associated with a positive result assessed on the ‘topically untreated side’, whereas the additional application of the new cream showed significantly greater improvement objectively measured by recognized methods for cellulite evaluation.

It is worth noticing that no restrictive diet was imposed in this study, as there was no calorie restriction and therefore no deprivation. Furthermore, diversity in meals is an important part of the success of the strategy to obtain the cooperation of the participants. The mean weight was 63.8 kg at baseline evaluation and 63.4 kg after 4 weeks: a very modest average reduction by 0.06%. The personal advice dispensed by an experienced nutritionist proved very helpful, but the topical application of this new specific cream produced even better significant results on clinical scoring – both visual and after pinching at T4W. Moreover, similar results were obtained after 4 weeks of treatment for the circumference measurement in centimetres of the upper part of the thigh and for the reconstructed volume measured by fringe projection.

The improvement in the quality of the skin is a very important factor in cellulite visibility. This was assessed through skin tonicity, which increased only on the treated side.

Most importantly, there is huge concurrence of the different types of cellulite evaluation in favour of the topically treated side. Consequently, we can conclude that this new topical treatment provides a genuine additional benefit to the dietary advice.

It would have been useful to evaluate the way people continue to apply the food recommendations 1 month after the end of the study by asking them to fill in the same questionnaire. As there is no deprivation in this balanced diet, we can assume that it is certainly easier for them to adhere to these recommendations over a long period of time. This, however, needs to be objectively proven. The long-term benefit of this complete treatment strategy, combining good food habits and an effective topical treatment, would be worth assessing.

In summary, we have shown that a personalized dietary advice to correct the ‘bad food habits’ had a slight but significant beneficial clinical effect on the cellulite clinical score on the upper part of each thigh after 4 weeks, whereas a highly significant difference in favour of the topically treated side was already recorded after 2 weeks. Moreover, whereas a slimming effect assessed by circumference measurement in centimetres of the upper thigh occurred after only 2 weeks of treatment on both thighs, at 4 weeks this reduction was significantly higher on the topically treated side than on the untreated side. This result corroborated the corresponding fringe projection volume measurements, which decreased significantly on the treated side only. The new topical cream definitely provides additional benefits in treating the cellulite appearance of the skin.
